# Nodal and BMP expression during the transition to pentamery in the sea urchin *Heliocidaris erythrogramma*: insights into patterning the enigmatic echinoderm body plan

**DOI:** 10.1186/s12861-017-0145-1

**Published:** 2017-02-13

**Authors:** Demian Koop, Paula Cisternas, Valerie B. Morris, Dario Strbenac, Jean Yee Hwa Yang, Gregory A. Wray, Maria Byrne

**Affiliations:** 1 School of Medical Science and Bosch Institute, The University of Sydney, Sydney, NSW 2006 Australia; 2 School of Life and Environmental Sciences, The University of Sydney, Sydney, NSW 2006 Australia; 3 School of Mathematics and Statistics, The University of Sydney, Sydney, NSW 2006 Australia; 4 Department of Biology and Center for Genomic and Computational Biology, Duke University, Durham, NC 27708 USA

**Keywords:** Sea urchin body plan development, Coelomogenesis, Deuterostome, Evolution, Direct development, Echinoid

## Abstract

**Background:**

The molecular mechanisms underlying the development of the unusual echinoderm pentameral body plan and their likeness to mechanisms underlying the development of the bilateral plans of other deuterostomes are of interest in tracing body plan evolution. In this first study of the spatial expression of genes associated with Nodal and BMP2/4 signalling during the transition to pentamery in sea urchins, we investigate *Heliocidaris erythrogramma*, a species that provides access to the developing adult rudiment within days of fertilization.

**Results:**

*BMP2/4*, and the putative downstream genes, *Six1/2*, *Eya*, *Tbx2/3* and *Msx* were expressed in the earliest morphological manifestation of pentamery during development, the five hydrocoele lobes. The formation of the vestibular ectoderm, the specialized region overlying the left coelom that forms adult ectoderm, involved the expression of putative Nodal target genes *Chordin*, *Gsc* and *BMP2/*4 and putative BMP2/4 target genes *Dlx*, *Msx* and *Tbx*. The expression of *Nodal*, *Lefty* and *Pitx2* in the right ectoderm, and *Pitx2* in the right coelom, was as previously observed in other sea urchins.

**Conclusion:**

That genes associated with Nodal and BMP2/4 signalling are expressed in the hydrocoele lobes, indicates that they have a role in the developmental transition to pentamery, contributing to our understanding of how the most unusual body plan in the Bilateria may have evolved. We suggest that the Nodal and BMP2/4 signalling cascades might have been duplicated or split during the evolution to pentamery.

**Electronic supplementary material:**

The online version of this article (doi:10.1186/s12861-017-0145-1) contains supplementary material, which is available to authorized users.

## Background

Despite the great diversity of animal body plans across invertebrate and vertebrate groups, the molecular mechanisms patterning the body axes that lie at the core of these plans are remarkably conserved [1–5]. For most Bilateria, the anteroposterior (A-P), dorsoventral (D-V) and left-right (L-R) axes are readily apparent, facilitating comparative studies of the molecular mechanisms patterning body axes and their evolution across divergent phyla [4]. Within the deuterostomes, however, the Echinodermata although initially bilateral as larvae, are pentameral as adults. This modification of the ancestral bilaterian plan makes identification of body axes for comparative studies problematic. The origin of the pentameral body plan thus becomes an important problem to solve because it is fundamental to understanding animal body-plan evolution, especially in a group so closely related to chordates [5, 6]. Whilst progress has recently been made in understanding the morphological development of pentamery and how the echinoderm body plan is related to the bilateral body plans of other deuterostomes [7, 8], the molecular mechanisms underlying the development of pentamery are not understood.

In vertebrates and invertebrates, Nodal and bone morphogenetic protein (BMP) signalling pathways are integral to embryonic axis determination [1–4, 9]. Nodal, a member of the TGFβ family of signalling proteins, plays a fundamental role in endomesoderm induction and in A-P and L-R axis formation [1, 2, 10]. BMP acts to organize body axes, by mediating D-V patterning and epidermal versus neural domains [4]. These signalling pathways have been a focus for evolutionary developmental biology in comparing the molecular mechanisms patterning body axes across the Metazoa and in identifying potential homologies [2, 4].

In echinoderms, the role of the Nodal and BMP signalling pathways in the development of axial features has been documented for the sea urchin echinopluteus larva [11–14]. Nodal patterns the aboral-oral (= pluteal D-V) axis in all three germ layers and the L-R axis [9, 10, 14]. Two organizing centers of *Nodal* expression have been proposed for these axes, one in the ventral ectoderm that organizes the D-V axis and one on the right side of the archenteron near its head that restricts the adult rudiment to the left side [9, 10, 15, 16]. Ectopic activation of Nodal results in a duplicate and fully patterned D-V axis in siamese plutei [16].

Expression perturbation experiments show the importance of Nodal and BMP signalling in regulating L-R axis development in sea urchin embryos and the interactions between Nodal and BMP and associated genes [12]. In sea urchin gastrulae, the *Nodal* expression in the right side of the archenteron is suggested to form an organizing center that propagates L-R asymmetry in a manner analogous to L-R organizing centers in vertebrates [10, 15]. Nodal regulates the transcription of *Nodal*, *Lefty* and *Pitx2* in the right ectoderm, and *Nodal*, *Not* and *Pitx2* expression in the right coelom, while BMP2/4 regulates transcription of *Six1/2* and *Eya* in the left coelom [10, 12, 15, 17, 18]. L-R asymmetry is also evident in the greater allocation of stem cells, the small micromeres, to the left coelom compared with the right coelom [19], a difference mediated by Nodal signalling and involved in formation of the adult body on the left side [12, 20].

The involvement of genes associated with the Nodal and BMP2/4 cascades in development of the pentameral, adult echinoderm body plan remain to be determined [17]. To address such possible involvement in patterning of the pentameral body plan, we used the sea urchin *Heliocidaris erythrogramma* as a model system that provides access to the adult rudiment within days of fertilization and for which a developmental transcriptome is available [21–23]. The morphogenetic mechanisms underlying coelom and juvenile rudiment development in *H. erythrogramma* are well described [24–31], providing the base for assessing gene expression patterns with respect to the development of pentamery. The pentameral plan develops from endomesoderm at the head of the archenteron [30]. The left coelom first forms as a single coelom and develops the five-lobed hydrocoele, which, lying at the core of pentamery, establishes the echinoderm body plan. The adult rudiment arises where the left coelom interacts with the overlying vestibular ectoderm, a specialized region that forms much of the adult ectoderm [29]. As the left coelom and vestibular ectoderm are key to the development of the adult sea urchin body plan, we focused on these structures.

To investigate the potential involvement of genes associated with the Nodal and BMP2/4 cascades based on previous studies of left-right specification in sea urchins, during the development of the pentameral body plan, we investigated the spatial localization of genes known to be expressed during rudiment formation in *H. erythrogramma* from recent transcriptome data [22]. Nodal associated genes were selected because they are involved in patterning mesoderm and endoderm in vertebrates [1, 2] and because of their role in patterning all three germ layers in the sea urchin pluteus [9, 10, 12, 15, 17, 18]. In addition, the location of *Nodal* expression in *H. erythrogramma* in the ectoderm and on the right side of the top of the archenteron is similar to that seen in other echinoids [27]. Functional studies show the importance of Nodal signaling in L-R axis formation in this species [27]. We also focused on BMP 2/4 because of its axis organizing role in bilaterian development and the importance of BMP in establishing left-side identity in sea urchins [4]. Two of the genes investigated, *Gsc* and *Msx*, are expressed in developing adult tissues in *H. erythrogramma* [25, 26]. Based on the transcriptome data [22], genes associated with Nodal and BMP2/4 signalling that had similar temporal expression profiles through juvenile development were investigated because of their possible regulatory role in morphogenesis of the adult body plan. We provide the first in situ hybridization, spatial expression data during sea urchin rudiment development for a suite of genes associated with Nodal and BMP2/4 signalling, specifically *Nodal*, *Lefty*, *BMP2/4*, *Chordin* and the putative downstream genes, *Pitx2*, *Gsc*, *Eya, Tbx2/3*, *Dlx*, *Six1/2* and *Msx*. Our results suggest that Nodal and BMP have a role in the transition to pentamery, contributing to our understanding of how the most unusual body plan in the Bilateria evolved.

## Results

### Morphology of *Heliocidaris erythrogramma* during adult rudiment formation

**Fig. 1:**
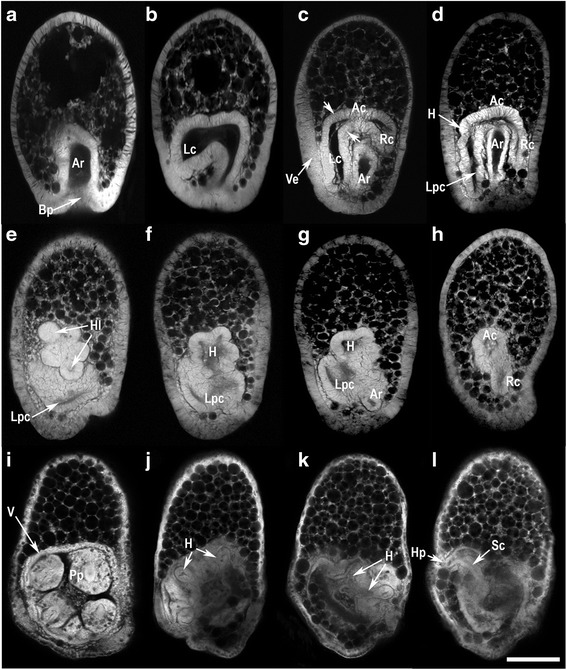
Confocal microscope sections of *Heliocidaris erythrogramma* from the gastrula to the rudiment stage larva. Orientation of larvae is with anterior to the top and posterior, the blastopore, to the base. The left coelom is either on the left (**a**–**d**), or the view is of the larval left side with the left coelom in frontal view (**e**–**l**). **a**–**b** Gastrulae with the archenteron and the left coelom. **c**–**d** Rudiment formation in the early larva begins with extension of the left and right coeloms posteriorly from the anterior coelom and the formation of the vestibular ectoderm. **e**–**h** Confocal sections through an advanced larva. Development of the anterior portion of the left coelom to form the hydrocoele lobes in the advanced larva. **i**–**l** Confocal sections through the same larva, the expansion of the hydrocoele lobes and the overlying vestibular ectoderm form the primary podia that are visible externally. The stone canal and vestibule are also evident. Ar, archenteron; Bp, blastopore; Lc, left coelom; Ac, anterior coelom; Rc, right coelom; Ve, vestibular ectoderm; Lpc, left posterior coelom; Hl, hydrocoele lobes; Pp, primary podia; V, vestibule; H, hydrocoele; Sc, stone canal; Hp, hydropore. Scale bar: 200 μm. See Morris [30] for a detailed assessment of coelomogenesis in *H. erythrogramma* though analysis of confocal sections

After gastrulation (Fig. 1a) the left coelom forms as a lateral out-pocketing of the anterior archenteron wall and extends posteriorly making contact with the left ectoderm (Fig. 1b). The right coelom begins to form. By 32 h post-fertilization (hpf), the left coelom has extended further posteriorly and extended the contact with the overlying ectoderm, the presumptive vestibular ectoderm that will invaginate during rudiment formation (Fig. 1c). This ectoderm is a morphologically distinct region composed of a pseudostratified epithelium. The left and right coeloms connect to the archenteron through the anterior coelom (Fig. 1d). The left coelom is partitioned into an anterior region that forms the hydrocoele and a posterior region that forms the left posterior coelom (= somatocoele) (Fig. 1d). The right coelom extends posteriorly but remains small (Fig. 1d). Invagination of the vestibular ectoderm begins around 36 hpf. Pentamery is evident in the five-lobed hydrocoele (Fig. 1e–g). The hydrocoele connects to the archenteron and right coelom at the anterior coelom (Fig. 1g, h). The hydrocoele lobes together with the ectoderm of the vestibule floor form the five primary podia (Fig. 1i–k). The stone canal connects the hydrocoele to the hydropore opening (Fig. 1l).

### Nodal and BMP expression during rudiment formation

#### *Nodal* and *Lefty*

**Fig. 2:**
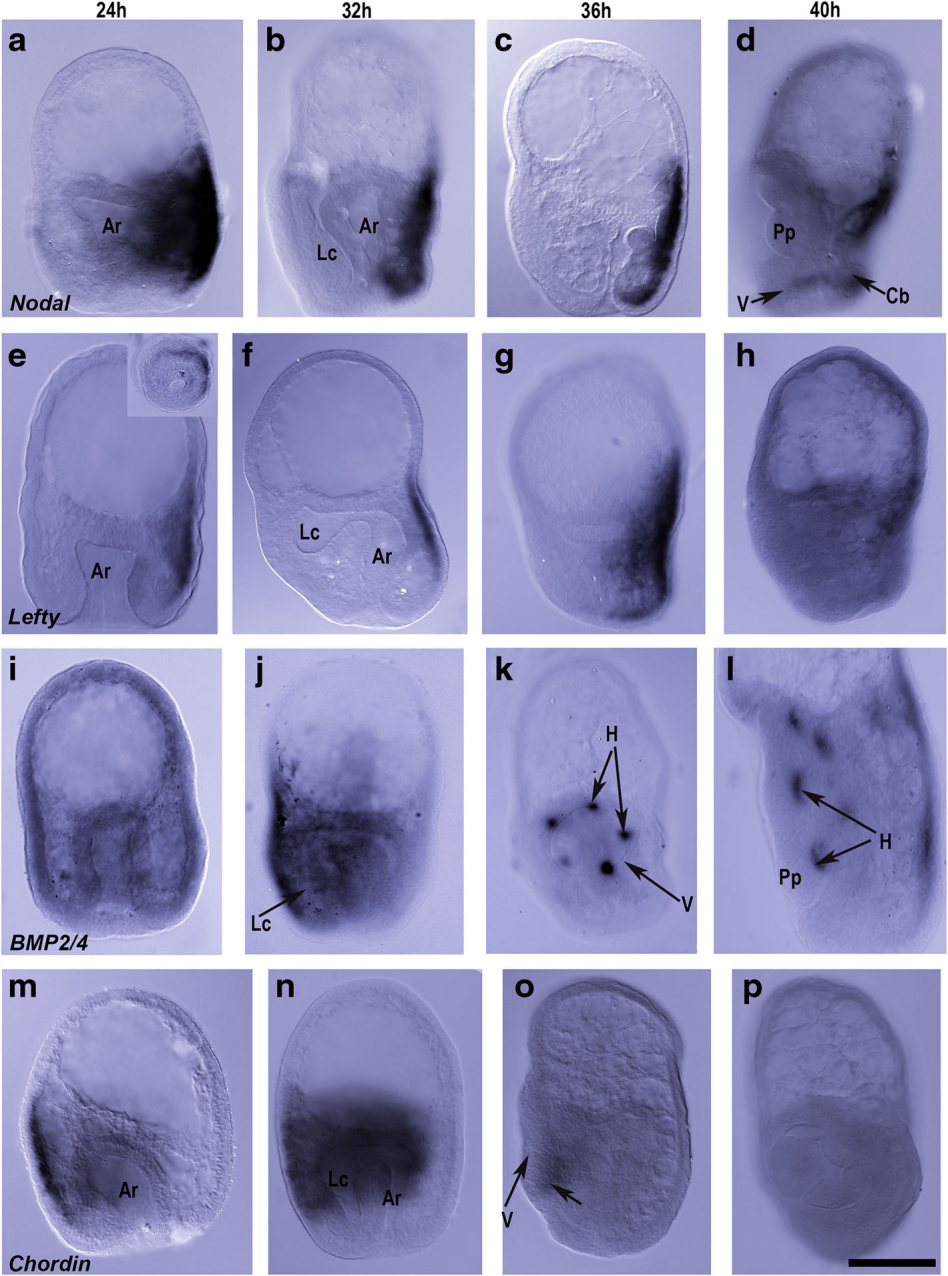
Expression of Nodal and BMP genes in *Heliocidaris erythrogramma* from gastrula to rudiment formation (24–40 hpf). Orientation of larvae is with anterior to the top and posterior, the blastopore, to the base. The left coelom is either on the left, or the view is of the larval left side with the left coelom in frontal view. **a**–**d**. *Nodal* was initially expressed in the right ectoderm extending approximately half way around the gastrula (**a**). This expression was reduced until only weak expression was detected along the right side and along the ciliated band of the 40 hpf larva (**d**). **e**–**h**. *Lefty* was also expressed in the right ectoderm extending halfway around the gastrula (**e**), (**e** insert, posterior view). *Lefty* was expressed in cells along the right ectoderm in a domain that was less extensive than *Nodal*, being reduced to a small domain at 40 hpf (**f**–**h**). **i**–**l**. *BMP2/4* was expressed in the posterior half of the gastrula ectoderm (**i**) and at 32 hpf *BMP2/4* was expressed in the presumptive vestibular ectoderm on the left side (**j**). A left-lateral view at 36 hpf (**k**) shows expression in the hydrocoele lobes with expression no longer detectable in the ectoderm. Left-lateral view of a 40 hpf larva (**l**) shows *BMP2/4* expression in five clusters of cells at the bases of the primary podia. **m**–**p** *Chordin* was expressed in the left ectoderm in the 24 hpf gastrula (**m**) and at 32 hpf (**n**) extends halfway around the larva. This is followed by weak (36 hpf, **o**) expression of *Chordin* in posterior portion of the vestibule and then no evidence of expression in the 40 hpf larva (**p**). Ar, archenteron; H, hydrocoele; Lc, left coelom; Pp, primary podia; Cb, ciliated band; V, vestibule. Scale bar: 200 μm

Both *Nodal* and its antagonist *Lefty* were expressed in the right ectoderm of the gastrula (Fig. 2a, e) with the *Nodal* expression being broader, extending further towards the left side than *Lefty*. As the left coelom formed and extended posteriorly (Fig. 1b, c), *Nodal* and *Lefty* at 32 h (Fig. 2b, f) remained expressed in the right ectoderm. By 36 hpf these genes were expressed in the right ectoderm with *Nodal* still more extensive than *Lefty* (Fig. 2c, g). With formation of the vestibule and the five primary podia, the expression of *Nodal* and *Lefty* was reduced (Fig. 2d, h), but *Nodal* remained expressed in a domain near the ciliary band and in the ectoderm overlying the anterior portion of the right coelom (Fig. 2d). *Lefty* was weakly expressed in the ectoderm overlying the anterior portion of the right coelom (Fig. 2h). The lower expression of *Nodal* from the gastrula (24 hpf) to the early rudiment stage larva (40 hpf) is consistent with temporal expression data (Additional file 1: Figure S1). While *Lefty* shares a similar temporal pattern, it appears to have its broadest domain of expression at 36 hpf.

#### BMP2/4

*BMP2/4* was initially expressed broadly in the ectoderm of the gastrula (24 hpf) (Fig. 2i). During formation of the left and right coeloms (Fig. 1b, c), the expression of *BMP2/4* at 32 hpf became localized to the left ectoderm in a domain corresponding to the presumptive vestibular ectoderm (Fig. 2j). As the vestibule and hydrocoele lobes formed, *BMP2/4* was no longer expressed in the ectoderm (Fig. 2k, l). By 36 hpf (Fig. 2k), there was a new domain of *BMP2/4* expression in the hydrocoele in five discrete domains associated with the developing hydrocoele lobes. The *BMP2/4* expression persisted in the developing hydrocoele as the rudiment formed. By 40 hpf (Fig. 2l), the *BMP2/4* expression was in the coelomic tissue layer at the bases of the primary podia.

#### Chordin

In the gastrula (Fig. 1a), the BMP antagonist *Chordin* was strongly expressed in the left ectoderm around half the gastrula (Fig. 2m). At 32 hpf (Fig. 2n) when the left coelom has formed and extended posteriorly, *Chordin* remained broadly expressed in the left ectoderm in a domain corresponding to the presumptive vestibular ectoderm extending partially around the larva. As the hydrocoele lobes formed (Fig. 1d), *Chordin* expression was reduced to a small region of ectoderm in the posterior vestibule (Fig. 2o). By 40 hpf (Fig. 2p), *Chordin* was no longer detected. The gradual reduction in expression was also evident in the temporal pattern of *Chordin* expression (Additional file 1: Figure S1).

### Downstream targets of Nodal and BMP are expressed in the left and right coeloms during rudiment formation

#### Pitx2

**Fig. 3:**
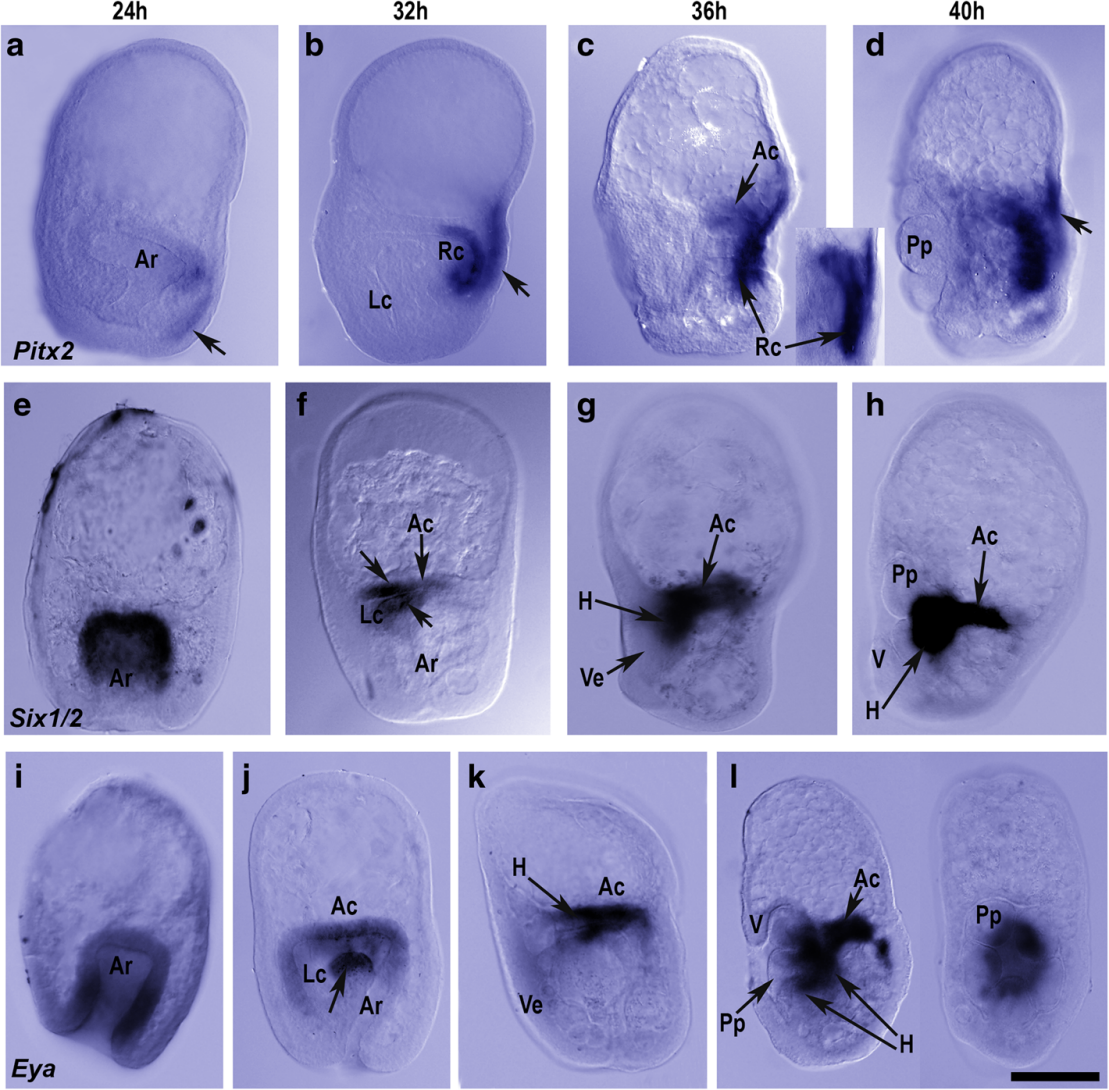
Expression of downstream targets of Nodal and BMP in the left and right coeloms during rudiment formation. Orientation of larvae is with anterior to the top and posterior, the blastopore, to the base and the left coelom on the left. **a**–**d** *Pitx2*. The gastrula (**a**) shows *Pitx2* expressed in the right side of the anterior archenteron where the right coelom is beginning to form and in the right posterior ectoderm. In the 32 hpf larva (**b**), *Pitx2* was expressed in the right coelom and right lateral ectoderm (*arrow*) and at 36 hpf (**c**) was expressed throughout the right coelom (insert) extending to where the anterior coelom meets the right coelom. In the 40 hpf (**d**) *Pitx2* was expressed in the right coelom and right ectoderm (*arrow*), with expression extending between the ciliated band and the lipid rich apical end of the larva. **e**–**h**. *Six1/2*. In the 24 hpf gastrula (**e**), *Six1/2* was expressed in anterior half of the archenteron. At 32 hpf (**f**) *Six1/2* was expressed in the anterior coelom at the head of the archenteron and in the anterior (*top arrow*) and posterior (*bottom arrow*) walls of the proximal left coelom. In the 36 hpf larva (**g**), *Six1/2* was expressed in the anterior coelom and hydrocoele underlying the vestibular ectoderm. The 40 hpf larva (**h**), shows *Six1/2* expression in the anterior coelom and hydrocoele. Expression is restricted to the central hydrocoele and does not extend into the lobes of the forming podia. **i**–**l**. In the 24 hpf gastrula (**i**), *Eya* was expressed in the mid region of the archenteron. In the 32 hpf larva (**j**), *Eya* was expressed at the head of the archenteron on the left side extending into the posterior wall of the proximal left coelom (*arrow*), as well as in the anterior coelom and the anterior wall of the proximal left coelom. In the 36 hpf larva (**k**), *Eya* was expressed in the anterior coelom and proximal hydrocoele. A lateral and frontal view of the adult rudiment in the 40 hpf larva (**l**) shows *Eya* expression in the anterior coelom, the hydrocoele and extending into the podia. Ar, archenteron; Lc, left coelom; Rc, right coelom; Ac, anterior coelom; Pp, primary podia; Ve, vestibular ectoderm; H, hydrocoele; V, vestibule. Scale bar: 200 μm

In gastrulae (Figs. 1a and 3a), *Pitx2* was expressed in the posterior ectoderm on the right side and in the right side of the anterior archenteron where the right coelom will form. At 32 hpf (Fig. 3b), as the left and right coeloms formed and extended posteriorly (Fig. 1c), *Pitx2* expression increased in the right coelom ending where it meets the anterior coelom. There was also *Pitx2* expression in the right ectoderm overlying the right coelom in a discrete anterior-posterior stripe (Fig. 3b), evident from an examination of the larva at different focal planes (not illustrated). As the hydrocoele lobes formed (Fig. 1e–g), *Pitx2* remained expressed throughout the right coelom and overlying right ectoderm (Fig. 3c). By the time the primary podia appeared (40 hpf) (Fig. 3d), *Pitx2* expression was more diffuse in the right ectoderm but remained strongly expressed in the ectoderm adjacent to the anterior end of the right coelom. Expression of *Pitx2* remained prominent in the right coelom (Fig. 3d).

#### Six1/2

In gastrulae, *Six1/2* was expressed in the anterior portion of the archenteron (Fig. 3e). This corresponds to the region from which the left and right coeloms will form (Fig. 1a, b,c). At 32 hpf (Fig. 3f), *Six1/2* expression was in the anterior coelom at the head of the archenteron and in the anterior and posterior walls of the proximal left coelom (see Fig. 1c). In 36 hpf larvae (Fig. 3g), expression of *Six1/2* was in the anterior coelom and in the hydrocoele. By 40 hpf (Fig. 3h), *Six1/2* was expressed in the anterior coelom and in the hydrocoele of the adult rudiment.

#### Eya

In 24 hpf gastrulae (Fig. 3i), *Eya* was expressed in the mid region of the archenteron. By 32 hpf (Fig. 3j), *Eya* was expressed in the anterior coelom and the left coelom, being especially strong in the posterior wall of the proximal left coelom that gives rise to the coelomic mesoderm. In 36 hpf larvae (Fig. 3k), *Eya* was expressed in the anterior coelom and in the proximal left coelom where the hydrocoele was forming. At 40 hpf (Fig. 3l), *Eya* was expressed in the anterior coelom and in the hydrocoele, extending into the podial lobes.

### Gene expression in the vestibule and pentameral structures

#### Gsc

**Fig. 4:**
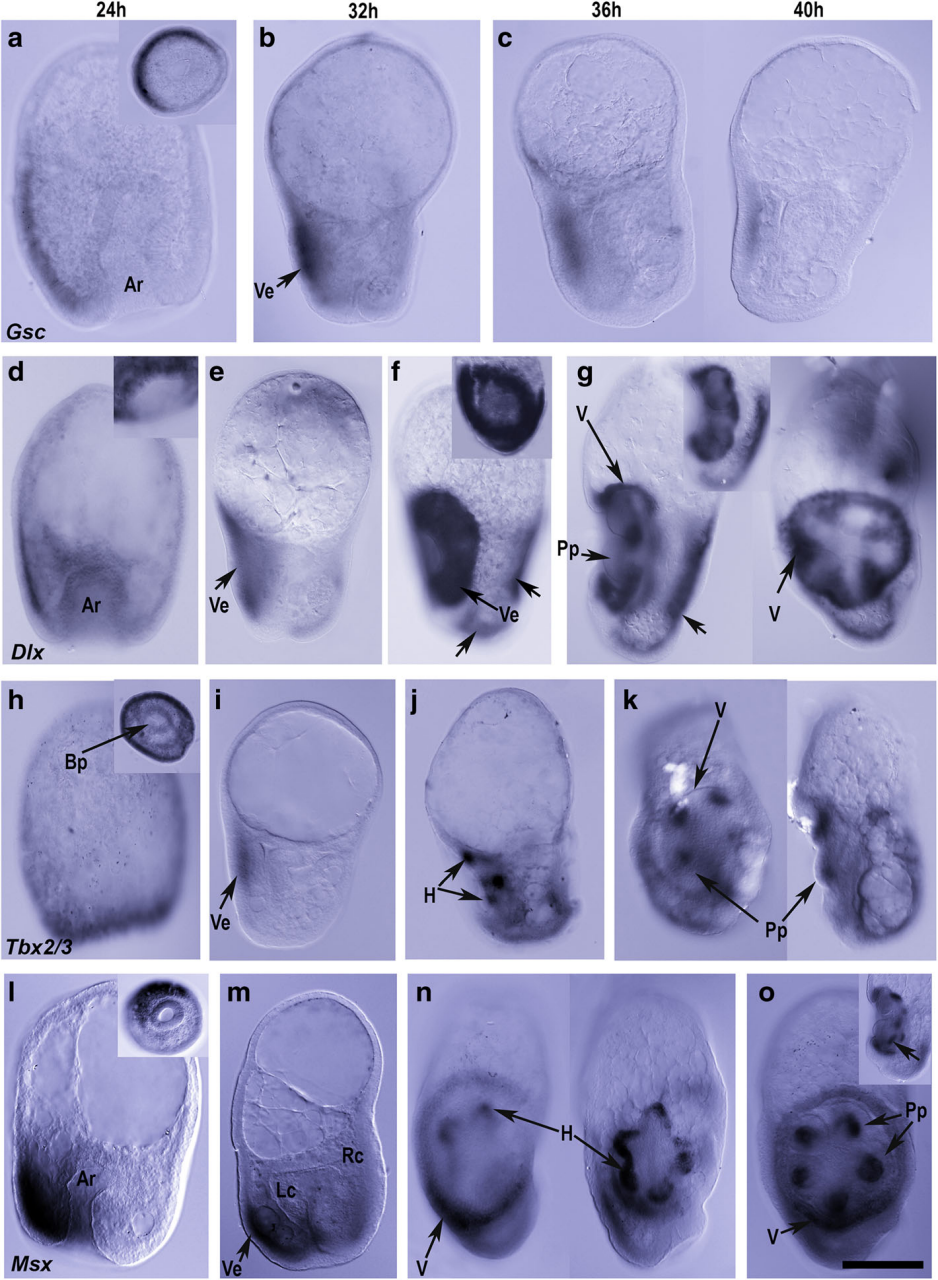
Expression in vestibular ectoderm and pentameral structures. Orientation of larvae is with anterior to the top and posterior, the blastopore, to the base. The left coelom is either on the left, or the view is of the larval left side with the left coelom in face view. **a**–**c** *Gsc*. At 24 hpf *Gsc* (**a**) was expressed in the posterior half of the gastrula ectoderm, extending halfway around as seen in the posterior view (insert). In the 32 hpf larva (**b**), *Gsc* was expressed on the left in the presumptive vestibular ectoderm. In the 36 and 40 hpf larvae (**c**) *Gsc* was expressed in the vestibular ectoderm. **d**–**g** *Dlx*. In the gastrula (24 hpf **d**), *Dlx* was expressed in the posterior ectoderm extending anteriorly to the head/tip/anterior of the archenteron. Expression extends halfway around the gastrula (insert). In the 32 hpf larva (**e**) *Dlx* expression was restricted to the vestibular ectoderm. At 36 hpf (**f**) expression was strong throughout the vestibular ectoderm with particularly strong staining around the rim of the vestibule (and insert). Additional expression was evident in the right and posterior ectoderm (*arrows*). In a lateral and frontal view of the adult rudiment in the 40 hpf larva (**g**), *Dlx* expression was in the ectoderm of the vestibule roof (insert shows a second plane of focus through vestibule) and in interambulacral regions of the vestibule floor ectoderm, as well as in the right ectoderm (*arrow*). **h**–**k** *Tbx2/3*. In the gastrula (**h**), *Tbx2/3* was expressed in the posterior ectoderm, close to the margins of the blastopore (insert, posterior view). In the 32 hpf larva (**i**), *Tbx2/3* was expressed in the vestibular ectoderm and at 36 hpf (**j**) was expressed in the hydrocoele lobes at the bases of the forming podia. A frontal and lateral view of the adult rudiment in the 40 hpf larva (**k**) shows *Tbx2/3* expression in the hydrocoele at the bases of the primary podia. **l**–**d** *Msx*. In gastrulae (**l**), *Msx* was expressed broadly in posterior ectoderm with stronger expression on the left side as also in the 32 hpf larva (**m**). In a left side view (two focal planes) of a 36 hpf larva (**n**) *Msx* was expressed in the rim of the vestibule, particularly in the posterior portion and in the hydrocoele lobes with strongest expression at the base of the lobes. In the left side view of a 40 hpf larva (**o**), *Msx* expression was evident in the posterior rim of the vestibule wall and in the hydrocoele at the bases of the primary podia (*arrow in insert*). Ar, archenteron; Lc, left coelom; Ve, vestibular ectoderm; V, vestibule; Pp, primary podia; Scale bar: 200 μm

In 24 hpf gastrulae (Figs. 1a and 4a), *Gsc* was initially expressed extensively around the greater part of the posterior ectoderm, approximately halfway around the gastrula, but excluded from the ectoderm around the blastopore, as described previously by Wilson et al. [25]. In 32 hpf larvae (Fig. 4b), *Gsc* was expressed in the left ectoderm in a domain corresponding to the presumptive vestibular ectoderm. At 36 hpf (Fig. 4c), *Gsc* was expressed in the vestibular ectoderm. By 40 hpf (Fig. 4c), *Gsc* was weakly detected in the vestibular ectoderm. This decline in expression was also evident in the temporal expression pattern of *Gsc* (Additional file 1: Figure S1).

#### Dlx

*Dlx* was expressed in the posterior ectoderm extending three quarters around the gastrula apparently centered on the left side (Fig. 4d). At 32 hpf (Fig. 4e), *Dlx* was expressed in the presumptive vestibular ectoderm and by 36 hpf (Fig. 4f), *Dlx* was strongly expressed in this region with the greatest expression around the rim of the vestibule (insert Fig. 4f). In addition, there was a domain of expression in the posterior and right ectoderm at both 36 and 40 hpf (Fig. 4f, g). By 40 hpf, *Dlx* was also expressed in the roof of the vestibule and in interambulacral domains of the vestibular floor (Fig. 4g).

#### Tbx2/3

In 24 hpf gastrulae (Figs. 1a and 4h), *Tbx2/3* was widely expressed in the posterior ectoderm close to the margins of the blastopore. By 32 hpf (Fig. 4i) expression became restricted to the left ectoderm in the presumptive vestibular ectoderm, particularly in its central region. By 36 hpf (Fig. 4j), expression was no longer detected in the vestibular ectoderm but a new domain of *Tbx2/3* expression was evident in distal ends of the hydrocoele lobes. At 40 hpf (Fig. 4k), *Tbx2/3* was strongly expressed in the hydrocoele lobes at the bases of the forming primary podia.

#### Msx

At 24 hpf (Fig. 4l), *Msx* was expressed in the posterior ectoderm around about half the gastrula assumed to be predominantly on the left side. By 32 hpf (Fig. 4m), the expression of *Msx* was detected largely in the posterior-most left ectoderm with weak expression extending toward the right side of the larva (see also [26]). In larvae with a vestibule (36 hpf, Fig. 4n), the expression on the left side of the larva was evident in the rim of the vestibule with strong expression in the posterior portion. Expression was also evident in a second domain in the hydrocoele, with strongest expression at the base of the lobes (Figs. 1f and 4n). In 40 hpf larvae (Fig. 4o), expression was still evident in the vestibule, in the roof just inside the rim, but it was weaker except in the posterior-most region. As the hydrocoele developed and the primary podia grew (Fig 1e, f), *Msx* expression appeared to be localized to the hydrocoele at the bases of the developing primary podia (Fig. 4o).

## Discussion

**Fig. 5:**
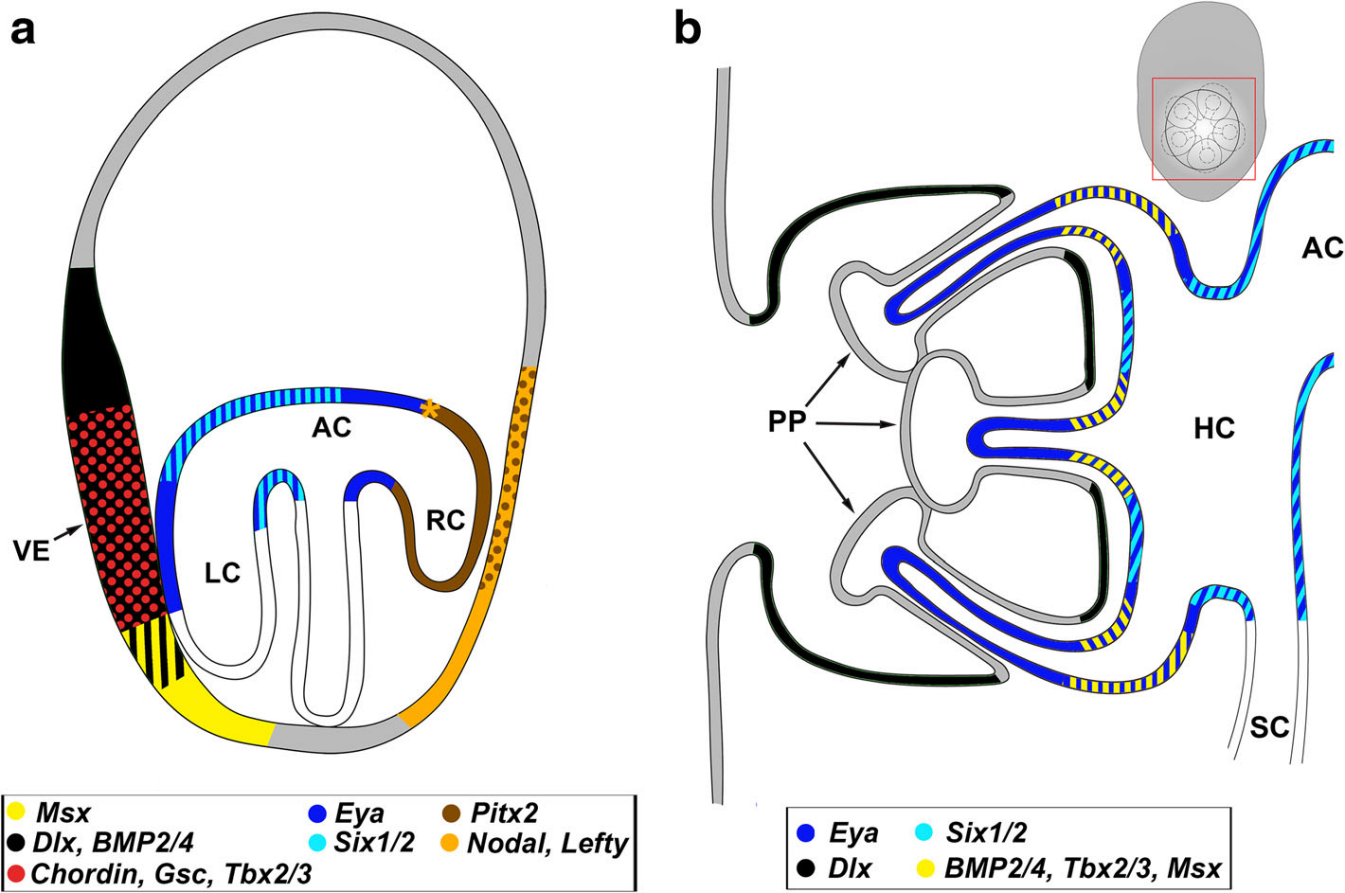
Diagrammatic representation of gene expression domains during the formation of the vestibular ectoderm and left coelom (**a**) at 32 hpf and the early rudiment (**b**) at 40 hpf. s. The left ectoderm adjacent to the left coelom thickens to form the vestibular ectoderm. The left and right coeloms have extended posteriorly, with the left coelom contacting the left ectoderm. The yellow asterisk indicates the location of *Nodal* expression to the right of the head of the archenteron in the developing right coelom of *Heliocidaris erythrogramma* (see [28]). This region may serve as an organizer analogous to that seen in vertebrates (see [3]). B. Sagittal view of the developing adult rudiment (region indicated in frontal view of the rudiment in insert). The hydrocoele and left posterior coelom (=somatocoele) are distinct and the hydrocoele lobes have extended to form the lumen of the primary podia. AC, anterior coelom; LC, left coelom; RC, right coelom; HC, hydrocoele; SC, somatocoele; VE, vestibular ectoderm; PP, Primary podia

In this first study of the genes associated with Nodal and BMP2/4 signalling during the transition to pentamery in sea urchins, we show that putative targets of Nodal and BMP2/4 are expressed in the pentameral hydrocoele and the vestibular ectoderm of *H. erythrogramma*, as summarised in Fig. 5a, b. That genes known to be downstream of Nodal (*BMP2/4*) and BMP2/4 (*Six1/2*, *Eya*, *Tbx2/3* and *Msx*) in early sea urchin development [12, 32], are expressed in the first morphological expression of pentamery, the five hydrocoele lobes, indicates that the Nodal and BMP2/4 signalling is likely to have a role in patterning the pentameral character of the echinoderm body plan (Fig. 5b). The formation of the vestibular ectoderm, the specialized region that overlies the hydrocoele lobes and which forms adult ectoderm, involves the expression of putative Nodal target genes, *Chordin*, *Gsc* and *BMP2/*4, and putative BMP2/4 target genes, *Dlx*, *Msx*, and *Tbx2/3* (Fig. 5a). The expression of *Nodal*, *Lefty* and *Pitx2* in the right ectoderm and *Pitx2* in the right coelom (Fig. 5a), is similar to that in development of other sea urchins [10, 12, 15, 17], where it has been attributed to patterning L-R asymmetry and restricting rudiment formation to the left side. In addition, negative regulators (e.g. *FoxQ2*, *Hbox12*, *Lefty*) also play key roles in modulating the location of nodal-expressing organizing centres by repressing *Nodal* expression in surrounding territories [33–35]. *Lefty*, an antagonist of *Nodal*, acts by diffusing farther through the embryo thereby limiting the territory of *Nodal* expression and may have a similar role in establishing the boundary of right sided *Nodal* expression as seen here for *H. erythrogramma* and in other sea urchins [12, 34–36].

As reported for other sea urchins [10, 15], *Nodal* expression is evident in the gastrula of *H. erythrogramma* at the head of the archenteron on the right side (see Smith et al. [28]) (Fig. 5a). This is similar in location to *Nodal* expressing cells in *S. purpuratus* and *P. lividus*, where they are suggested to function as an endomesoderm organizer *sensu* development in other Bilateria [10]. *Nodal* expression in the right ectoderm of *H. erythrogramma* (Fig. 5a) is also as in other sea urchins where it is suggested that reciprocal signalling between endomesoderm and ectoderm is important in determination of L-R asymmetry [15]. In *H. erythrogramma* there is no indication of a second centre expression of *Nodal* as seen in the ventral ectoderm in gastrulae of species with indirect development where Nodal functions to patterning the D-V axis [10, 16]. *Heliocidaris erythrogramma* lacks the clear dorsal-ventral ectodermal domain of the echinopluteus [37].

The *Nodal* signalling centre at the head of the archenteron on the right side (see Smith et al. [28]) might pattern the development of coelomic mesodermal structures in the late gastrula. In *H. erythrogramma, Six1/2* and *Eya* were expressed in the anterior and left coeloms and remained expressed in these domains during the posterior extension of the left coelom (Fig. 5a). *Six1/2* was expressed in the proximal left coelom (Fig. 5a). *Eya* was strongly expressed in the posterior wall of the proximal left coelom (Fig. 5a), a region that possibly gives rise to the coelomic mesoderm [8].

Nodal at the head of the archenteron is also likely to be involved in the later transition to pentamery. The earliest pentameral pattern of gene expression observed during *H. erythrogramma* development was the expression of *BMP2/4*, *Msx* and *Tbx2/3* at 36 hpf in the hydrocoele lobes. A pentameral expression of *Eya* and *Six1/2* was observed later at 40 hpf in the primary podial lobes. At 40 hpf, *BMP2/4*, *Msx* and *Tbx2/3* were still expressed in a pentameral pattern in coelomic tissue at the bases of the primary podia (Fig. 5b). This region corresponds to the postulated growth zone at the bases of the primary podia [38]. *Msx* injected into *H. erythrogramma* eggs results in hypertrophic development of the primary podia [25], suggesting *Msx* has a role in their growth. The pentameral expression pattern of *BMP2/4* cascade genes at the outset of hydrocoele development, the core of the pentameral adult body plan, and in growth of the primary podia indicates a potential role for this signalling system in the initiation and continued development of these pentameral structures.

The vestibular ectoderm, a specialised thickened region of pseudostratified epithelium which overlies the developing left coelom, and later forms most of the adult ectoderm also appears to be patterned by Nodal and BMP signalling. In *H. erythrogramma*, *Chordin*, *Gsc* and *BMP2/4*, all known targets of Nodal in early sea urchin development [12, 14, 15, 31, 32], are initially expressed broadly in the left ectoderm and become restricted to the vestibular ectoderm (Fig. 5a) [26]. Interestingly, two other known targets of Nodal, *Bra* and *Not1* [12, 14, 15, 32] are expressed in the vestibular ectoderm of *S. purpuratus* [39]. Although *Chordin* was not detected in the early larva of *S. purpuratus* [12], we expect that *Chordin* is involved in the formation of the vestibular ectoderm in other sea urchin species, since adult rudiment development in pre-metamorphic larvae is likely to be conserved. The first pentameral expression detected in the vestibular ectoderm was for *Dlx*, a putative target of BMP2/4, in the interambulacral domains at 40 hpf.

Evidence that patterning of the vestibular ectoderm involves an intra-ectodermal mechanism is provided by microsurgery experiments with *H. erythrogramma* [40, 41]. Isolated ectodermal shells generated by removal of the archenteron and developing coeloms in gastrulae (16–24 hpf) develop normal vestibular ectoderm on the left side [41, 42]. The autonomous production of vestibular ectoderm in isolated ectoderm indicates that the formation of this domain does not require signals from the left coelom [29]. Based on our data, the gastrulae that were operated on would have expressed *Nodal* in right ectoderm and *BMP2/4* broadly in the ectoderm. We suggest that the intra-ectodermal mechanism specifying vestibular ectoderm (*sensu* [29]), involves the expression of genes downstream of Nodal that become restricted to the left (*Chordin*, *Gsc, BMP2/*4) or right (*Nodal*, *Pitx, Lefty*) ectoderm (Fig. 5a). The expression of putative *BMP2/4* target genes (*Msx*, *Tbx2/3*, *Dlx*) and the BMP antagonist *Chordin* in the left ectoderm suggests that BMP signalling in the left ectoderm may have a fine-tuning role in establishing the vestibular ectoderm domain (Fig. 5a). Intra-ectodermal signalling of BMP may differentiate vestibular and non-vestibular ectoderm on the left side. Thus, right-sided Nodal signalling may restrict vestibular ectoderm formation to the left, while BMP signalling on the left may be responsible for fine-tuning of the position of the vestibule. This hypothesis needs to be addressed through functional investigations.

An interaction between the vestibular ectoderm and the mesoderm in the development of the definitive juvenile is clear from explant studies, which demonstrate that coelomic tissue induces neural and podial ectoderm in the vestibule floor [41]. The signalling mechanisms involved in this interaction are poorly understood. Based on the disruption of development in *H. erythrogramma* using NiCl_2_, Minsuk et al., [29] suggest that pentameral patterning is influenced by a permissive signal from the ectoderm. The presence of *BMP2/4* in presumptive vestibular ectoderm of *H. erythrogramma* prior to the appearance of the pentameral hydrocoele indicates that BMP signalling may be involved.

While it appears that the BMP cascade is involved in patterning the developing hydrocoel and thus formation of the sea urchin adult body plan, the molecular events involved in the transition to pentamery prior to anatomical evidence of the five-lobed hydrocoele are not known. The spatial expression pattern of several genes (*BMP2/4*, *Dlx*, *Tbx2/3* and *Msx*) in development of pentameral structures in *H. erythrogramma* suggests the involvement of a gene regulatory network (GRN) similar to the one that is active during early embryonic development in sea urchins [10, 23, 32, 43]. Development of pentamery may involve discrete five-fold expression of genes in the endomesoderm at the head of the archenteron before pentamery is evident. In this scenario, the evolution of pentamery may have involved a duplication or a split in a signalling cascade during evolution of pentamery. If pentamery arose through duplication [31, 38] then the formation of the five lobed hydrocoele may indicate the presence of five signalling centres. Temporal expression data shows that *Nodal* expression in *H. erythrogramma* is high through initial development of the hydrocoele (to 40 hpf) and *BMP2/4* expression continues through advanced rudiment development (40–96 hpf) [22]. As *BMP2/4* is known to be downstream of Nodal signalling in early sea urchin development [32], and is temporally expressed through rudiment formation, then a duplication or split may have involved the BMP2/4 cascade. Support for this suggestion would require investigation of the Nodal-BMP GRN during the transition to pentamery.

In *H. erythrogramma*, a species where a feeding larva does not intervene between gastrulation and adult body plan development, genes associated with Nodal and BMP2/4 signalling are expressed from the gastrula to rudiment development [22]. It is not known if the Nodal-BMP network is also active throughout development in sea urchins that have a feeding larva or if there is a hiatus in this network between the feeding larval stage and rudiment development. The marked compression in timing and rewiring of the GRN in direct development in *H. erythrogramma*, compared with that in indirect developers [23] indicates a major change in gene expression in evolution of development. The temporal pattern of gene expression during the transition to pentamery remains to be determined for species with a feeding larva.

Candidate cells involved in the generation of pentamery in sea urchins are the small micromeres seen in species with indirect development. Different R-L allocation of the small micromeres to the developing coeloms is mediated by Nodal signalling and is involved in formation of the adult body on the left side [12, 20]. During gastrulation the small micromeres become positioned at the head of the archenteron and, in most cases, five are allocated to the left coelom and three to the right coelom [12, 19]. The left coelomic pouch is where the descendants of the small micromeres accumulate [44]. *Nodal* and *Pitx2* are expressed in the small micromeres that become positioned in the right coelom (but not those on the left) and Nodal signalling in the right coelom leads to apoptosis of these cells [12]. Thus, only the five small micromeres in the left coelom survive to contribute to development of the adult and are the source of the germ line. An intriguing possibility is that these five cells may also be involved in setting up pentamery, a suggestion that would need to be addressed through lineage analysis of their descendants. However, ablation of these cells does not interfere with development of the rudiment, although the resulting adults lack germ cells [20]. *H. erythrogramma* and other direct developing echinoids have equal cleavage and so lack small micromeres. While there are no data on similar germ line stem cells or molecular markers (eg. *vasa*) in development of these echinoids, a search for germ cell determinants may provide a clue to the development of pentamery.

Due to the conserved role of the Hox cluster in animal body axis formation throughout the Bilateria, several studies of development of the adult echinoderm body plan have focused on these genes to address questions on the evolution of pentamery (review [7]). While these studies provide important information on the expression of these genes in echinoderm development, there is little indication that Hox genes are the source of pentamery [7]. In consideration of the roles that Nodal and BMP2/4 signalling systems are likely to play in patterning pentamery, we suggest that the pentameral expression of Hox and a plethora of other genes (e.g. [45]) are 5-fold iterations in an existing pentameral organization, and are not involved in setting up the pentamery.

## Conclusions

In conclusion it appears that the Nodal and BMP signalling cascade which plays highly conserved, roles in patterning animal development [46–49] has been modified in echinoderms to generate evolutionary novelty in a pentameral body plan. However, many questions remain. For instance, perturbation studies are needed to determine the extent to which BMP signalling is responsible for patterning the vestibular ectoderm. The well-characterized Nodal-BMP GRN provides a powerful tool to identify candidate gene regulatory interactions in development of pentamery. Of particular interest is to determine whether the expression of Nodal- and BMP-associated genes during juvenile development is regulated by the same inputs from the GRN that are active during early development [10, 18, 23], or whether a distinct set of inputs is used to regulate their expression during patterning of the juvenile. Candidate gene interactions need to be tested with targeted functional analyses such as morpholino knock-downs. Furthermore, other signalling cascades such as the Hedgehog and WNT are likely to be involved in patterning the early rudiment and contributing the establishment of pentamery [17]. *Heliocidaris erythrogramma* is an ideal subject for carrying out such studies, due to the ease of rearing many individuals through metamorphosis and their robustness to in situ hybridization protocols.

## Methods

### Animal culture and sample collection

*Heliocidaris erythrogramma* collected from Little Bay, New South Wales (33°58′S, 151°14′E) were placed in aquaria at ambient temperature (20–21 °C). Spawning was induced by injection of 0.5 M KCl and the gametes were used to establish embryo cultures using routine methods. Each fertilization used the gametes of 2–3 males and females and the embryos were reared at 20–21 °C. Developmental stages were collected at four time points, gastrula (24 h post fertilization - hpf), early larva with developing coeloms (32 hpf), mid stage larva with the hydrocoele (36 hpf) and the early rudiment larva (40 hpf) (Fig. 1). These time points match those for which we have transcriptome data (JA Wygoda, Y Yang, M Byrne and GA Wray [21]. The stages investigated cover the period from the development of left and right structures of the larva to the development of the oral and aboral structures of the adult (Fig. 1) (see [30, 50]).

### Whole mount in situ hybridization

For whole mount in situ hybridization (WMISH) specimens were fixed in 4% (w/v) paraformaldehyde in DEPC-treated filtered seawater (FSW 1.0 μm Millipore) and dehydrated through a graded series of methanol to 100%. They were then stored in 100% methanol at -20 °C until use for WMISH.

The spatial expression of eleven genes (*Nodal, Lefty, BMP 2/4, Chordin, Gsc, Tbx2/3, Pitx2, Eya, Msx, Six1/2, Dlx)* was investigated. cDNA was synthesised using the Superscript III Reverse Transcriptase (Invitrogen) and gene specific primers (Additional file 2: Table S1) that were designed based upon homologous sequences obtained from the *H. erythrogramma* transcriptome [21]. Amplified fragments were cloned into pGEM-T vector (Promega) and sequenced to confirmed gene identity. WMISH followed the methods of Byrne et al., [51]. Probe detection was carried out using NBT/BCIP (Roche) in a detection buffer containing 10% polyvinyl alcohol. Reactions were stopped with several rinses in sterile water. Specimens were then dehydrated in ethanol, cleared in benzyl benzoate/benzoic acid (1:2) and photographed using a DF13 camera mounted on a BX60 microscope (Olympus). The colour reaction times used were chosen to optimize the signal to noise ratio. These differed depending on developmental stage where background signal varied largely due to the size and location of lipid reserves.

Orientation of larvae in the WMISH figures is with anterior to the top of the figure and posterior, the blastopore, to the base. The left coelom is either on the left, or the view is of the larval left side with the left coelom in face view. L-R larval axes and oral-aboral adult axes are coincident.

### Confocal microscopy

Larvae were fixed for confocal microscopy in 2.5% (v/v) glutaraldehyde (ProSciTech, Australia) in FSW for 1–2 h, washed in FSW, dehydrated in an ethanol series to 70% (v/v) ethanol in Milli-Q water and stored at 4 ^o^C. For the observations, larvae were dehydrated to 100% ethanol and cleared in 2:1 (v/v) benzyl benzoate/benzyl alcohol and viewed in this clearant after mounting in a coverslip-sealed chamber. The larvae were autofluorescent after the glutaraldehyde fixation. The larvae were viewed in an Olympus (Japan) FluoView 1000 laser scanning system (version 1.7.1.0) linked to an Olympus IX81 inverted microscope. Each specimen was excited at λ_ex_633 nm with a helium–neon laser and detected at λ_em_645–745 nm.

The larvae in the confocal sections (Fig. 1) are oriented with anterior to the top and posterior, the blastopore, to the base. The views are of the larval dorsal side with the left coelom on the left of the section, or they are of the larval right side, which is an aboral view of the adult.

Detailed assessments of the morphology of embryo and larval development with a focus on coelomogenesis in *H. erythrogramma* though analysis of sections are available in the studies of Smith et al. [27, 28] and Morris [30].

## Additional files

Additional file 1: Figure S1. Temporal expression profile of 11 Nodal-BMP associated genes of *Heliocidaris erythrogramma* at four time points in development. The temporal expression profiles of the 11 genes investigated in this study, derived from the developmental transcriptome of *Heliocidaris erythrogramma*. Includes methods of analysis. (PDF 2472 kb)
Additional file 2: Table S1. Primers used to isolate gene orthologs from *Heliocidaris erythrogramma*. A list of primer sequences used in this study to isolate orthologous genes from *Heliocidaris erythrogramma*. (DOCX 15 kb)

## Abbreviations

A-P: Anteroposterior
D-V: Dorsoventral
hpf: Hours post-fertilization
L-R: Left-right
