# Supplementary material for: Nodal and BMP expression during the transition to pentamery in the sea urchin Heliocidaris erythrogramma: insights into patterning the enigmatic echinoderm body plan
Source: BMC Dev Biol. 2017 Feb 13;17:4. doi: 10.1186/s12861-017-0145-1 (PMC5307799; doi:10.1186/s12861-017-0145-1)
Supplement: Additional file 1: Figure S1. — Temporal expression profile of 11 Nodal-BMP associated genes of Heliocidaris erythrogramma at four time points in development. The temporal expression profiles of the 11 genes investigated in this study, derived from the developmental transcriptome of Heliocidaris erythrogramma. Includes methods of analysis. (PDF 2472 kb) [file 12861_2017_145_MOESM1_ESM.pdf]

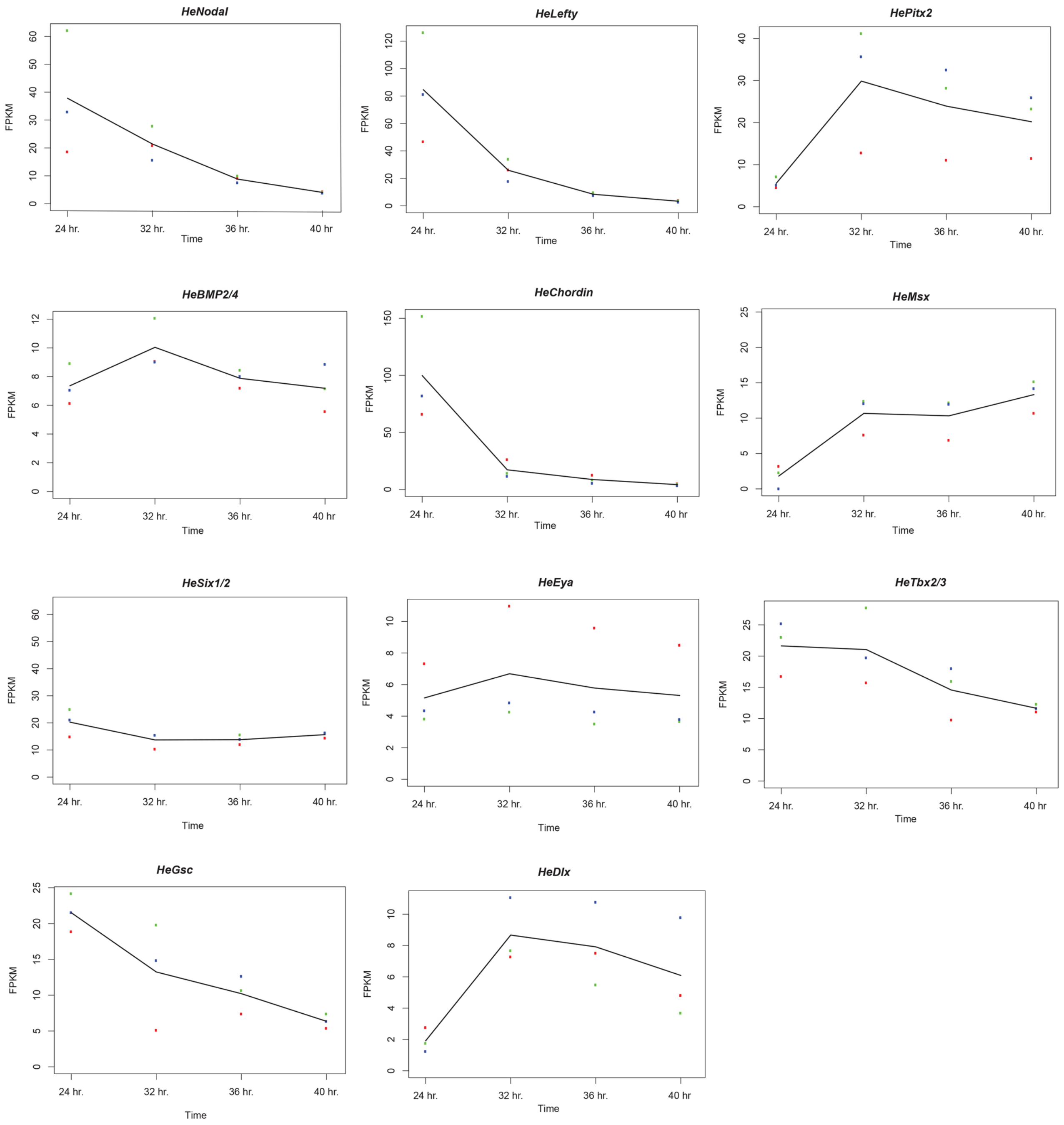

Fig. S1. Temporal expression profile of 11 Nodal-BMP associated genes of *Heliocidaris erythrogramma* at four time points in development.

Expression profiles of 11 genes investigated in this study determined from the *H. erythrogramma* developmental transcriptome [1]. Library construction, sequencing and de novo transcriptome assembly are detailed in [1]. Read quantification, association and analysis of temporal expression are described in [2]. The genes were selected based on their asymmetric ectodermal and coelomic expression in *Paracentrotus lividus* and *Strongylocentrotus purpuratus* [3-8] and their relationship based on transcriptomic analysis of Nodal and BMP associated genes in *H. erythrogramma* [2]. Transcripts of *Nodal*, *Lefty*, *BMP2/4*, *Chordin*, *Pitx2*, *Msx*, *Eya*, *Six1/2* and *Tbx 2/3* were identified using command line nucleotide BLAST version 2.2.28 [9]. The e-value cut-off used was 1 x 10^{-20}. The Fragments per kilobase of transcript per million mapped read (FPKM) expression values are plotted for the four developmental stages covered in this study from gastrula (24 hpf) to the early rudiment larva (40 hpf). Each stage has three replicates. Line plots were made with R version 3.0.3 [10].

References

1. Wygoda JA, Yang Y, Byrne M, Wray GA: **Transcriptomic analysis of the highly derived radial body plan of a sea urchin**. *Genome Biology and Evolution* 2014, **6**(4):964-973.
2. Byrne M, Koop D, Cisternas P, Strbenac D, Yang JYH, Wray GA: **Transcriptomic analysis of Nodal-and BMP-associated genes during juvenile development of the sea urchin Heliocidaris erythrogramma**. *Marine genomics* 2015, **24**:41-45.
3. Molina MD, de Crozé N, Haillot E, Lepage T: **Nodal: master and commander of the dorsal–ventral and left–right axes in the sea urchin embryo**. *Current opinion in genetics & development* 2013, **23**(4):445-453.
4. Lapraz F, Besnardeau L, Lepage T: **Patterning of the dorsal-ventral axis in echinoderms: insights into the evolution of the BMP-Chordin signaling network**. *PLoS Biol* 2009, **7**(11):e1000248.
5. Luo Y-J, Su Y-H: **Opposing Nodal and BMP signals regulate left–right asymmetry in the sea urchin larva**. *PLoS Biol* 2012, **10**(10):e1001402.
6. Duboc V, Röttinger E, Lapraz F, Besnardeau L, Lepage T: **Left-right asymmetry in the sea urchin embryo is regulated by nodal signaling on the right side**. *Developmental Cell* 2005, **9**(1):147-158.
7. Bessodes N, Haillot E, Duboc V, Röttinger E, Lahaye F, Lepage T: **Reciprocal signaling between the ectoderm and a mesendodermal left-right organizer directs left-right determination in the sea urchin embryo**. *PLoS Genetics* 2012, **8**(12):e1003121.
8. Saudemont A, Haillot E, Mekpoh F, Bessodes N, Quirin M, Lapraz F, Duboc V, Rottinger E, Range R, Oisel A *et al*: **Ancestral regulatory circuits governing ectoderm patterning downstream of Nodal and BMP2/4 revealed by gene

regulatory network analysis in an echinoderm. *PLoS Genetics* 2010, **6**(12):e1001259.

9. Camacho C, Coulouris G, Avagyan V, Ma N, Papadopoulos J, Bealer K, Madden TL: **BLAST+: architecture and applications**. *BMC Bioinformatics* 2009, **10**:421.

10. Team RC: **R: A language and environment for statistical computing**. In.: R Foundation for Statistical Computing, Vienna, Austria; 2012.
