# Supplementary material for: Nodal and BMP expression during the transition to pentamery in the sea urchin Heliocidaris erythrogramma: insights into patterning the enigmatic echinoderm body plan
Source: BMC Dev Biol. 2017 Feb 13;17:4. doi: 10.1186/s12861-017-0145-1 (PMC5307799; doi:10.1186/s12861-017-0145-1)
Supplement: Additional file 2: Table S1. — Primers used to isolate gene orthologs from Heliocidaris erythrogramma. A list of primer sequences used in this study to isolate orthologous genes from Heliocidaris erythrogramma. (DOCX 15 kb) [file 12861_2017_145_MOESM2_ESM.docx]

**Table S1** Primers used to isolate gene orthologs from *Heliocidaris erythrogramma*. Primers were designed using the longest obtainable sequence for the gene of interest from the *H. erythrogramma* transcriptome database. RT-PCR was used and amplified fragments were cloned into a pGEM-T vector (Promega) and sequenced to confirm identity.

| **Gene** | **Primer** | **Sequence** |
| --- | --- | --- |
| *Nodal* | Forward | CTCCCATCATCAGCCATCGG |
|  | Reverse | TGCCCAATCCATATGAGCGT |
| *Lefty* | Forward | ATGGCCGAACTCAAAGTCTAC |
|  | Reverse | GGAACTCCTGTCCCTTATATGC |
| *BMP2/4* | Forward | GGTTCCACATCATCCTCTACATC |
|  | Reverse | CGGCCATCATCGCTAAACA |
| *Chordin* | Forward | CGCACCTACCCAGACGATAC |
|  | Reverse | AACGATGCGTCCACGTAACT |
| *Pitx* | Forward | ACCCATCGACAGTACCCCTC |
|  | Reverse | GGAGAACGTCGAACTGAGCG |
| *Distalless (dlx)* | Forward | CCCCGATATACAACCATTCG |
|  | Reverse | TAGGGGTGGGGTGTAGATGA |
| *Tbx2/3* | Forward | TGATTCGGACGATCCTCAAGTT |
|  | Reverse | ATCCATCCTCCTCTCGTGGT |
| *Msx* | Forward | GCGTTGGCGAAGAAATAAGG |
|  | Reverse | CAGCACCCGACAGATATGG |
| *Gsx* | Forward | ACCTCTAACTCCAACCAACG |
|  | Reverse | GTGATGAGGGTGGTGTCC |
| *Eya* | Forward | CCAATGGACTCGGAAGAGG |
|  | Reverse | GGTGGTTCTCAGAGGTATCC |
| *Six1/2* | Forward | CCCTAAATTACAGGCTCTCTGG |
|  | Reverse | CCAACGTGTGCTAAGACTAGG |
